# Abstract of the Proceedings of the Erie County Medical Society

**Published:** 1868-06

**Authors:** M. G. Potter

**Affiliations:** Sec’y.


### Abstract of the Proceedings of the Erie County Medical Society.

The semi-annual meeting of the Erie County Medical Society was held in Buffalo on the 9th instant. The following gentlemen were elected to membership on compliance with the by-laws:—Drs. Eddy, Hopkins, Schuyler, Willoughby, Nichols and Chace, of Buffalo; Dr. W. D. Murray of Tonawanda, and Dr. Newman B. L. Parker of Akron.

The literary exercises consisted of an oration by Dr. Henry Lapp of Clarence, and a memoir of the late Dr. Cyrenius Chapin by Dr. G. F. Pratt of Buffalo. After the applause which followed the reading of Dr. Pratt's paper, Dr. James P. White said in substance, as follows:

I have been more than gratified in listening to the memoir that has been read in our hearing. The excellencies of the pioneers of our profession in Western New York, are indeed well calculated to fill our hearts with pride. The record of these excellencies, unless preserved for us in papers like the one to which we have listened, must soon be beyond our reach. The lives of such men should not thus cease to exert their influence upon us. One such memoir, that of Dr. Marshall, has been deposited in the archives of this Society, and permit me to express the hope that this Society may have, and urge its right to have, and that at no distant day another memoir of our honored dead from the pen of his surviving son. No man is so fitly prepared to perpetuate the memory and influence of my departed friend, Dr. Trowbridge, and none more willing to do us this high honor.

After other appropriate remarks by Drs. Boardman and Snow, it was, on motion of Dr. James P. White,

*Resolved*, That the thanks of this Society be tendered Dr. Pratt for his very interesting memoir; that at the expense of this Society 1000 copies be published in pamphlet form for gratuitous distribution, and 600 copies for distribution with the Buffalo Medical Journal, and that a copy be presented to the State Medical Society, accompanied with a request for its publication.

The committee appointed to select orators for the meeting in January next, reported the names of Dr. William C. Phelps for orator, and Dr. C. F. A. Nichell for substitute.

M. G. POTTER, Sec'y.

